# Timescales for detecting a significant acceleration in sea level rise

**DOI:** 10.1038/ncomms4635

**Published:** 2014-04-14

**Authors:** Ivan D. Haigh, Thomas Wahl, Eelco J. Rohling, René M. Price, Charitha B. Pattiaratchi, Francisco M. Calafat, Sönke Dangendorf

**Affiliations:** 1 Ocean and Earth Sciences, National Oceanography Centre, University of Southampton, European Way, Southampton SO14 3ZH, UK; 2 School of Civil, Environmental and Mining Engineering and the UWA Oceans Institute, The University of Western Australia, 35 Stirling Highway, Crawley, Western Australia 6009, Australia; 3 Research Centre Siegen, University of Siegen, Hagener Street 139, 57072 Siegen, Germany; 4 College of Marine Science, University of South Florida, 140 7th Avenue South, St. Petersburg, Florida 33701, USA; 5 Research School of Earth Sciences, The Australian National University, Canberra 0200, Australia; 6 Department of Earth and Environment and SERC, Florida International University, 11200 SW 8th Street, OE-148, Miami, Florida 33199, USA; 7 National Oceanography Centre, Waterfront Campus, European Way, Southampton SO14 3ZH, UK; 8 Research Institute for Water and Environment, University of Siegen, Paul-Bonatz-Street 9-11, 57076 Siegen, Germany

## Abstract

There is observational evidence that global sea level is rising and there is concern that the rate of rise will increase, significantly threatening coastal communities. However, considerable debate remains as to whether the rate of sea level rise is currently increasing and, if so, by how much. Here we provide new insights into sea level accelerations by applying the main methods that have been used previously to search for accelerations in historical data, to identify the timings (with uncertainties) at which accelerations might first be recognized in a statistically significant manner (if not apparent already) in sea level records that we have artificially extended to 2100. We find that the most important approach to earliest possible detection of a significant sea level acceleration lies in improved understanding (and subsequent removal) of interannual to multidecadal variability in sea level records.

## Abstract (web summary)

Global sea levels are rising as a result of climate change, but at what rate, and whether this rate is increasing is open to debate. Haigh *et al.* show that the earliest detection of significant increase in the rate of sea level rise can only be achieved once interannual and multidecadal variability is removed.

## Graphical abstract

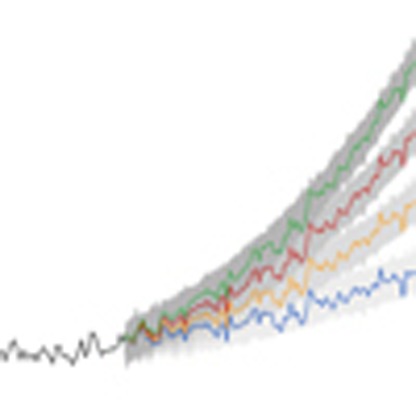

A rise in global sea level is one of the most certain consequences of climate change. The Intergovernmental Panel on Climate Change’s (IPCC) Fifth Assessment Report (AR5)^1^ projected a global sea level rise of 0.28–0.98 m for 2100, compared with 1986–2005. The lower end is broadly consistent with linear continuation of the average rate of rise observed over the 20th century^2^, while the upper value (and larger projected rises^3^,^4^,^5^,^6^,^7^) requires a significant increase (that is, an acceleration) on the average 20th century rate.

Earliest possible detection of a significant increase in the rate of sea level rise is helpful to inform the public and support direct political action to enable adequate adaptation^8^, particularly for projections with higher rates of rise. However, while we are virtually certain, based on proxy^9^,^10^ and instrumental data^2^,^11^,^12^,^13^,^14^, that the rate of global sea level rise has increased during the last two centuries (from relatively low rates of change in the order of tenths of mm per year during the late Holocene, to modern rates in the order of mm per year), a consensus has yet to be reached about the existence and significance of any further acceleration in recent years, which would be indicative of a high sea level projection pathway.

Instead of trying to determine whether the rate of sea level rise is increasing further and, if so, by how much, we here introduce a different and novel approach. We ask when accelerations might become apparent for different sea level projections (if not apparent already). First, we apply the two main methods that have been used previously for the detection of accelerations in the historical data, to identify accelerations in the sea level records artificially extended to 2100. We discuss several pitfalls in using these acceleration detection methods and demonstrate the invalidity of several arguments used to suggest that the rate of sea level rise is not currently increasing. Second, we use our results to discuss why considerable debate seems to unnecessarily persist around the topic. In particular, we examine the important issue of sea level variability, the presence of which makes it difficult to assess whether accelerations are due to natural internal climate variability or anthropogenic causes. We repeat our analysis, using sea level records adjusted to account for natural internal climate variability, and in the process demonstrate that the most important approach to earliest possible detection of a significant increase in the rate of sea level rise lies in improved understanding (and subsequent removal) of interannual to multidecadal variability in sea level records.

## Results

### Sea level records artificially extended to 2100

**Figure 1:**
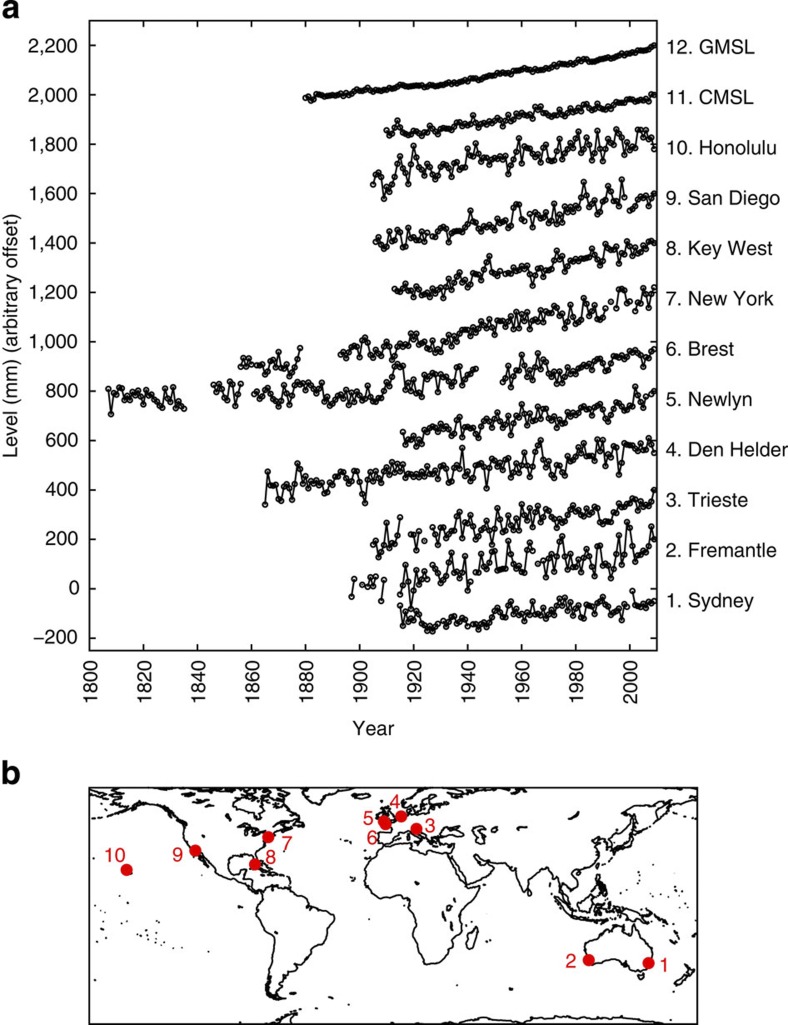
Mean sea level time series. (**a**) The 12 (10 tide gauge records, the CMSL and the GMSL) annual mean sea level records used in the present study, offset (by 200 mm) for clarity of presentation; (**b**) location of the 10 tide gauge sites.

**Figure 2:**
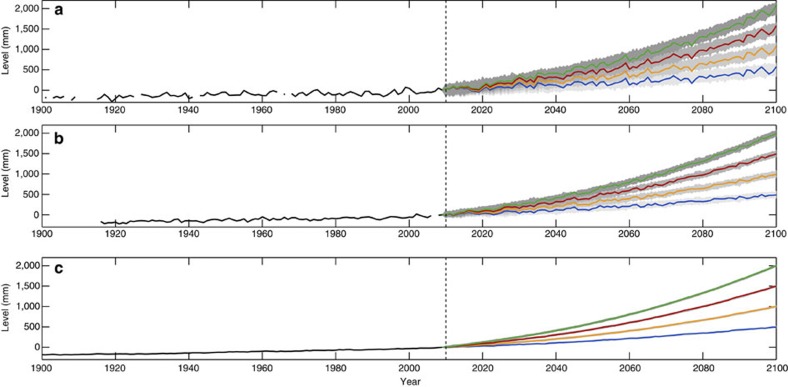
Sea level time series artificially extended to 2100. (**a**) Fremantle; (**b**) Newlyn; and (**c**) the GMSL record. For the period from 2010–2100, the coloured lines (blue for 0.5 m of sea level rise by 2100; orange 1 m, red 1.5 m and green 2 m) show only 1 of the 10,000 randomly generated time series. The grey shaded areas (with the grey scale varied for each of four sea level projections) show the envelope for all 10,000 randomly generated time series.

We focus on 12 sea level records (see Methods and Table 1); 10 individual tide gauge records, a coastal mean time series and a global sea level reconstruction (Fig. 1). The coastal mean is an approximation based on a simple average of the 10 tide gauge records (hereafter ‘coastal mean sea level’ (CMSL)) and the other is the sophisticated reconstruction from Church and White^2^ (hereafter ‘global mean sea level’ (GMSL)). To cover the most commonly reported estimates^1^,^3^,^4^,^5^,^6^,^7^,^15^, we consider four sea level projections to the target year 2100. These correspond to 0.5 and 1 m (approximately mid and upper AR5 range^1^), and 1.5 and 2 m (upper end of range suggested by refs 3, 4, 5, 6, 7) of sea level rise by 2100 (hereafter P1, P2, P3, P4, respectively). Using each of the 12 records and each of the four sea level projections, we create time series that artificially extend the 12 records to 2100. These comprise the following: prior to 2010, the specific historic record; and from 2010–2100, one of the four sea level projections superimposed with realistic interannual variability, which we randomly generate using a noise model^16^ with autocorrelation and variance parameters obtained from the relevant historic records (Fig. 2). To account for uncertainty in the timing of future interannual variability, we create 10,000 randomly generated noise time series (see Methods), for each of the 12 sea level records in turn, and superimpose these on each of the four sea level projections from 2010–2100.

### Acceleration detection technique 1

The question whether the rate of sea level rise has increased has most often been addressed by adding a quadratic term to the linear regression model and estimating its value and uncertainty, using either individual tide gauge records^17^,^18^,^19^,^20^,^21^,^22^, or global reconstructions^2^,^11^,^12^,^13^,^23^,^24^. This is the first acceleration detection technique that we consider. Our main issues with this approach, as others (for example, refs 25, 26, 27) have highlighted before, is that: first, the actual year at which a significant acceleration is first identified depends strongly on the start date, time period and the length of the time period of the sea level record for which the quadratic coefficient is estimated; and, second, quadratic equations can be a poor fit to observed sea level change, because of the considerable natural internal and anthropogenic variability evident in sea level records over a range of timescales.

To illustrate this, we estimate quadratic coefficients (and hence accelerations) and their uncertainty (95% confidence) for the 12 sea level records considered here, for different historic periods (Tables 1, 2). Of the complete sea level records available, only the three longest records (New York, Brest and GMSL) have an acceleration significantly different from zero (Table 1), consistent with results from other studies (for example, refs 14, 17, 18, 21). However, when accelerations are estimated for just the periods 1880–2009 and 1900–2009, then the accelerations are no longer significantly different from zero at New York and Brest (Table 2). Only the GMSL retains an acceleration significantly different from zero for the period 1915–2009 when data are available for all sea level records, allowing direct comparison. For the period 1930–2009, none of the 12 records has an acceleration significantly different from zero (except Brest), in general agreement with results from the controversial study of Houston and Dean^21^. The acceleration at Brest is different from Newlyn, despite their close proximity, and may appear significantly different from zero because of a data gap in the 1940s. This highlights another problem with assessing sea level accelerations (that is, missing data). Missing data can also introduce spurious accelerations in averages of tide gauge records and also in sea level reconstructions that use a time-varying tide gauge distribution. Four of the records (Newlyn, Brest, Trieste and CMSL) have accelerations significantly different from zero for the period 1960–2009.

The contrasts in both sign and magnitude of the estimated acceleration between the 12 records, and for different periods, highlight the challenges in comparing results among studies that focus on sea level records with varying start dates and which cover different periods. The contrasts also highlight the danger of choosing one particular start date (for example, 1930 used by ref. 21) to confirm an argument (for example, ‘the rate of sea level rise is not increasing’), while ignoring a significant portion of data that may contradict that position.

**Figure 3:**
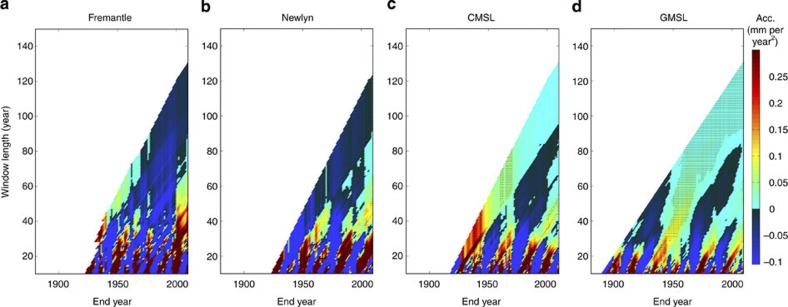
Accelerations for historic sea level records. (**a**) Fremantle to illustrate a site with relatively large interannual variability; (**b**) Newlyn to illustrate a site with relatively small interannual variability; (**c**) the CMSL time series, created by averaging the 10 tide gauge records; and (**d**) the GMSL record. Hatched area identifies plot regions where accelerations are significantly different from zero (95% confidence interval).

To avoid bias, we therefore recommend, and here use, the approach of Jevrejeva *et al.*^12^,^24^, (also applied in refs 25 and 26), which systematically estimates quadratic coefficients and their uncertainty (95% confidence) for all possible start dates and data lengths of our artificially extended records (see Methods). For just the historic records, our results (Fig. 3a,b), like ref. 26, show that accelerations (that is, two times the quadratic coefficient) are rarely diagnosed to be statistically different from zero in individual tide gauge records, particularly in records shorter than about 130 years. In tide gauge records, both the magnitude and sign of the acceleration are dominated by interannual to multidecadal variability: to a greater extent for sites with large variability such as Fremantle; and to a lesser but still significant extent for sites with smaller variability such as Newlyn. Note that large accelerations observed over shorter timescales are real. However, they are mainly due to natural internal variability and mask any externally forced accelerations (see Discussion).

**Figure 4:**
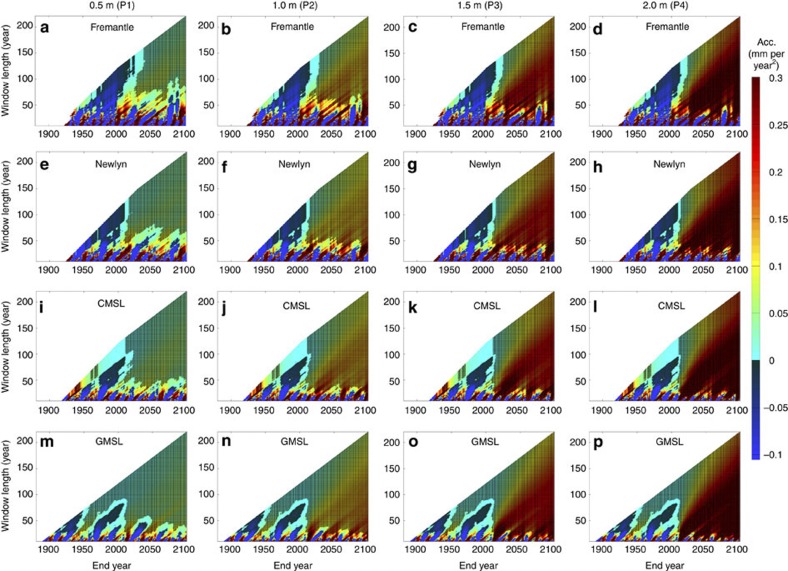
Accelerations for sea level records artificially extended to 2100. (**a**–**d**) Fremantle to illustrate a site with relatively large interannual variability; (**e**–**h**) Newlyn to illustrate a site with relatively small interannual variability; (**i**–**l**) the CMSL time series, created by averaging the 10 tide gauge records; and (**m**–**p**) the GMSL record. Hatched area highlights plot regions where accelerations are significantly different from zero (95% confidence interval). Note, for each sea level record, the plots show results for just one of the 10,000 randomly generated noise time signals.

For the records artificially extended to 2100, our results (Fig. 4a–h) suggest that accelerations statistically different from zero are only likely to consistently become evident in tide gauge records (irrespective of start dates) as late as the 2030s, for sea level rise pathways towards lower targets of 0.5–1 m (P1, P2) (if interannual and multidecadal variability is not accounted for, see Discussion). For sea level rise pathways towards upper targets of 1.5–2 m by 2100 (P3, P4), accelerations statistically different from zero are likely to consistently become evident in tide gauge records during the 2020s (again if variability is not taken into account).

Research has often focused on global reconstructions (for example, refs 2, 11, 12, 13, 23), because global sea level has an order of magnitude smaller internal variability than sea level at individual sites^2^. For the CMSL (Fig. 3c) and GMSL (Fig. 3d) records, we find that the sign of the acceleration alternates between positive and negative for different start dates, when curves are fitted to periods shorter than about 90 years, indicative of a clear influence of multidecadal variability. However, over longer periods, positive accelerations statistically different from zero are consistently evident in the GMSL record and are likely to steadily increase in magnitude over the remainder of the 21st century (Fig. 4m–p). Positive accelerations are also evident, over longer periods, in the CMSL record, but these are not currently statistically different from zero (if interannual to multidecadal variability is not taken into account, see Discussion). For the CMSL records artificially extended to 2100, our results (Fig. 4i–l) suggest that accelerations statistically different from zero are likely to become evident later this decade for all four sea level projections. Note that all significant (95% confidence) accelerations observed in Figs 3, 4 are positive for window lengths greater than about 40 years, and that no significant deceleration is ever detected for any combination of window length and end year.

Houston and Dean^21^ argued that there is a lack of evidence for the accelerations that would be necessary to achieve the upper end of the IPCC projected range^15^ because the acceleration observed in the GMSL record^2^ and in long tide gauge records is an order of magnitude smaller than the required rates (~0.1 mm per year^2^). Our results (Fig. 4) clearly demonstrate that accelerations are not expected to exceed 0.1 mm per year^2^ until the second half of the 21st century for sea level rise pathways towards targets of 0.5–1 m (P1, P2), and will only exceed this threshold around 2030–2050 for pathways towards targets of 1.5–2 m (P3, P4). Thus, our analysis implies that the argument presented by Houston and Dean^21^ is invalid. In fact, by simply visually inspecting the projections from the earlier IPCC Third Assessment Report and Fourth Assessment Report (AR4), it is clear that only small rates of acceleration were predicted by the IPCC models for the period from 1990–2010. Hunter and Brown^28^ calculated an average acceleration in the central projection of the IPCCs AR4 A1FI emission scenario (including scaled-up ice sheet discharge) of 0.002 mm per year^2^ over the period 1990–2010 (see the value plotted at 2000 in their Fig. 1, ref. 28), which agrees closely with observations from altimetry and GMSL reconstructions, over this period. The recent projections, from the IPCCs AR5 representative concentration pathway (RCP) 8.5 (which we use here, see Methods), very closely resemble quadratic curves and have near constant accelerations of ~0.064, 0.096 and 0.136 mm per year^2^ over the period 1990–2100, for the lower, central and upper projection range, respectively. These accelerations are larger than the acceleration observed in the altimetry and GMSL reconstruction over the period 1990–2010, but are still within the (66% confidence) uncertainty range (see Table 1 in ref. 28). Therefore, it is intriguing that arguments persist that because only small accelerations are presently evident, the IPCC sea level projections must be wrong, when in fact the observations over the last 20 years agree closely with the Third Assessment Report and AR4 projections and are statistically consistently with AR5 RCP8.5 projections. Further, as we showed above, it will take time before accelerations that exceed 0.1 mm per year^2^ are detected for the upper RCP8.5 projection (that is, P2).

### Acceleration detection technique 2

Next, we consider the implications of another commonly used approach. The question whether the observed high rates of sea level rise of the last two decades^2^,^29^,^30^,^31^ represent a significant and sustained acceleration has been regularly evaluated by estimation of linear rates for consecutive overlapping periods. This method has been applied widely, using both tide gauge records (for example, refs 32, 33, 34, 35, 36, 37) and global reconstructions (for example, refs 2, 11, 12, 13), with the paper by Holgate^33^ being one of the most cited examples. Studies that have applied this technique have (with the exception of ref. 37) inferred that the high rates of rise observed over the last two decades are not significantly larger than rates observed at other times within the past two centuries. That result has led some authors, notably Houston and Dean^21^,^38^, to argue that recent high rates might simply result from interannual to multidecadal variability, and hence to infer that there would be no evidence that sea level rise is following a high projection pathway.

The estimation of linear rates for consecutive overlapping periods is the second acceleration detection technique that we consider. The key weakness of this approach has already been demonstrated by Rahmstorf *et al.*^39^ Using essentially the approach we emulate and extend here (that is, a synthetic sea level time series, consisting of a smooth sea level rise plus artificially generated noise), they illustrated how the derivative of ‘noisy’ data is invariably more ‘noisy’ (see their Fig. 3 in ref. 39). Thus, even a small amount of noise in a sea level record obscures the acceleration signal when looking at decadal rates of rise. The pertinent question for our assessment with this method therefore is when we might (or more fundamentally should) expect to detect rates that are significantly higher than past rates for different sea level projections, given this key weakness of the method. (Note that because of these problems, like Rahmstorf *et al.*^39^, we advocate the use of low pass filtering techniques, such as those previously applied by Wahl *et al.*^34^,^40^ to detect changes in observed sea level records that deviate from a simple straight line or a quadratic curve).

In this assessment, we fit linear regressions over overlapping periods of different lengths to each of our artificial time series, and estimate the value and uncertainty (at 95% confidence) of the linear term for each period. For each artificially extended time series, we identify the end year of the period when the linear term for that particular period is (and remains) statistically higher than all the linear terms estimated for periods of the same length in the historic pre-2010 period. We also take into account the uncertainty due to future interannual variability (see Methods).

**Figure 5:**
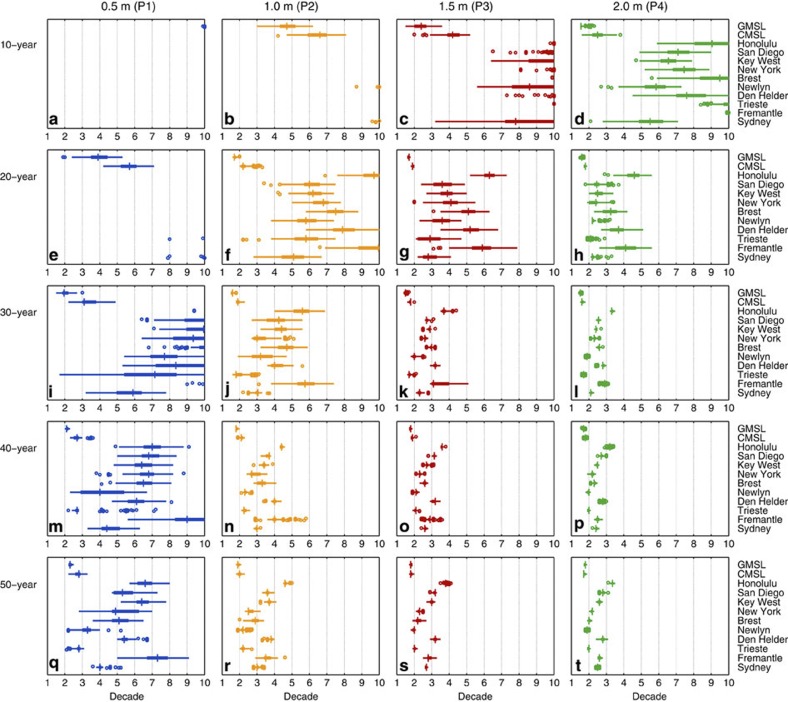
Years when unprecedented linear rates of sea level rise are first identified. Linear rates have been estimated for 10- (**a**–**d**), 20- (**e**–**h**), 30- (**i**–**l**), 40- (**m**–**p**) and 50-year (**q**–**t**) consecutive overlapping periods and for projections of 0.5 (P1), 1 (P2), 1.5 (P3) and 2 m (P4) of sea level rise by 2100. For each record and projection, box plots indicate the range of years identified for the 10,000 synthetic time series. On each box, the central mark is the median, the edges of the box are the 25th and 75th percentiles, the whiskers extend to the most extreme data points not considered outliers and outliers are plotted individually as circles. Values along the *x* axes indicate the decade within the 21st century (1 is 2010, 2 is 2020, and so on).

We detect linear rates that are significantly higher than past rates (that is, unprecedented rates) earliest when 30- to 40-year overlapping periods are used, and much later when shorter (10- to 20-year) periods are considered (Fig. 5). This is a particularly important finding, because previous authors (for example, refs 2, 11, 12, 13, 32, 33, 34, 35, 36, 37) tended to estimate linear rates for 10- to 20-year overlapping periods, to match the length of altimetry data available at the time of their analysis. Crucially, if sea level follows P1, an unprecedented rate of rise is unlikely to be detected using 10-year periods until after 2100 for each of the 12 records (Fig. 5a), because of the considerable interannual to multidecadal variability present in the records, and for P2 only after 2030 using the GMSL record (Fig. 5b). Holgate^33^ used 10-year overlapping periods, and our analysis reveals that that data length, on the sites considered, is not suited for the detection of unprecedented linear rates of sea level rise during the 21st century (for the range of sea level projections considered here), because of the considerable variability (see Discussion).

We find that unprecedented linear rates are detected earliest, in most records, when 40-year overlapping periods are used (Fig. 5m–p). Using 40-year overlapping periods on the GMSL record, we identify rates of sea level rise that are significantly higher than past rates in the mid to late 2010s for a rise towards 1.5–2 m (P3, P4), and late 2010s to early 2020s for a rise towards 0.5–1 m (P1, P2). Relative to this, the CMSL record reveals unprecedented rates up to 2 years later for P3 and P4 and up to 5 years later for P1 and P2. Individual tide gauge records only reveal an unprecedented rate up to 25 years later than GMSL for P3 and P4, and as much as 60 years later for P1 and P2.

Our analysis thus disproves arguments (for example, refs 21, 38) that unprecedented rates of sea level rise should have been detected by now if sea levels were currently following a high projection pathway. Instead, rates significantly higher than past rates are only likely to become detectable later this decade, or early next decade, in the CMSL and GMSL data sets, and up to 60 years later in individual tide gauge records (if interannual and multidecadal variability is not taken into account, see Discussion).

## Discussion

Our examination of sea level accelerations in a future context highlights that considerable debate (about the existence of a significant and sustained increase in the rate of sea level rise in recent years) persists mainly because different studies have used different acceleration detection methods, on different sea level records and/or subsets of these records. Those studies have typically been done: without considering the appropriateness of the selected acceleration detection methods; without systematic investigation of mutual consistency between different detection methods; without a detailed assessment of sensitivity of the estimated acceleration to start date, time period and data length of the records analysed; and most importantly, without accounting for interannual to multidecadal sea level variability.

The issue of variability is particularly important: first, because the presence of considerable variability, especially on a local scale, makes it difficult to assess whether observed changes are due to natural or anthropogenic causes^26^,^27^,^41^; and second, because the presence of considerable natural and anthropogenic variability means that the observed sea level curve is not adequately represented by a simple linear or quadratic model (see also ref. 25). Hence, it can lead to estimates of rates of change that, while real on a local scale, mostly reflect internal natural variability, rather than providing evidence for, or against, externally forced accelerations. In studies of global sea level change^2^,^11^,^12^,^13^,^23^,^24^, reconstruction techniques are often used to account for internal variability and as a result, interannual and multidecadal sea level variations are much weaker in GMSL records. Therefore estimates of accelerations in these time series are more robust^27^. However, GMSL reconstructions still show significant temporal variability because of the following: the poor spatial sampling of the tide gauge data set used; the way in which the tide gauge record distribution changes over time; the fact that sea level variability at tide gauge sites is often dominated by coastal processes that are not captured by satellite altimetry; and the still relatively short temporal coverage of altimetry data^27^.

**Figure 6:**
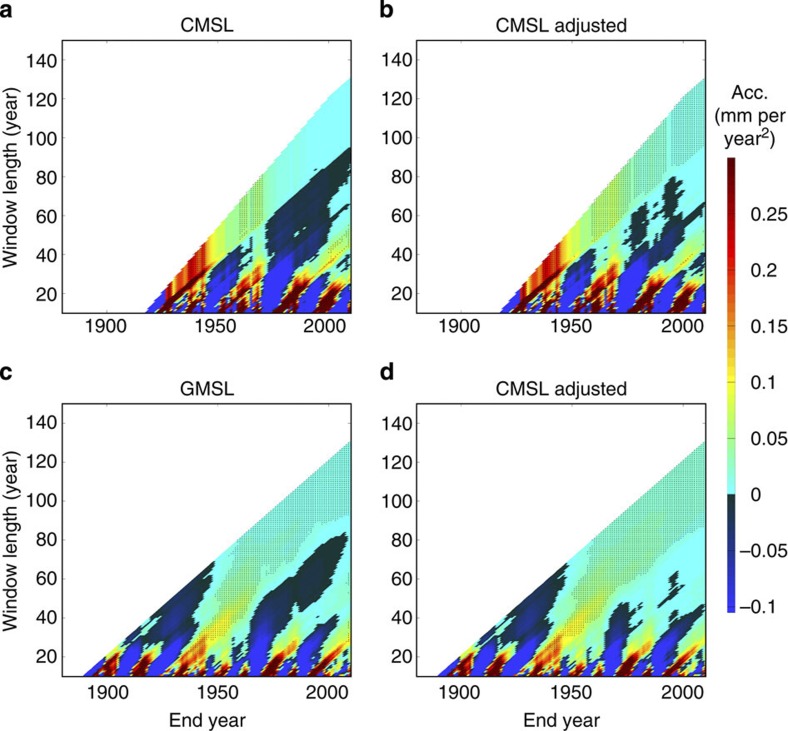
Accelerations for historic sea level records. (**a**) The CMSL, created by averaging the 10 tide gauge records; (**b**) the CMSL after adjusting for reservoir impoundment using estimates from Chao *et al.*^42^; (**c**) the GMSL; and (**d**) the GMSL after adjusting for reservoir impoundment, again using the estimate from Chao *et al.*^42^ Hatched area identifies plot regions where accelerations are significantly different from zero (95% confidence interval).

Interannual to multidecadal sea level variations are primarily caused by natural internal climate variability (for example, changes in atmospheric forcing and related ocean mass or heat redistribution, arising for instance from regional climate influences such as El Niño–Southern Oscillation or the North Atlantic Oscillation^26^,^27^, or volcanic eruptions^42^), but can also arise due to non-climatic influences, such as the increase in water storage behind man-made dams since the 1950s^43^. Corrections can be, and have been, made (for example, refs 2, 5, 25) for the water storage component. Doing so using estimates from ref. 43 (Fig. 6), we obtain accelerations in the adjusted CMSL record that are currently statistically different from zero (Fig. 6b), and we identify significant accelerations in the adjusted GMSL record earlier (Fig. 6d). But it is clear that internal climate variability also needs to be taken into account to allow better assessment of any acceleration over the last century that can be attributed to the causes other than natural variability, and to allow detection of such accelerations as early as possible.

**Figure 7:**
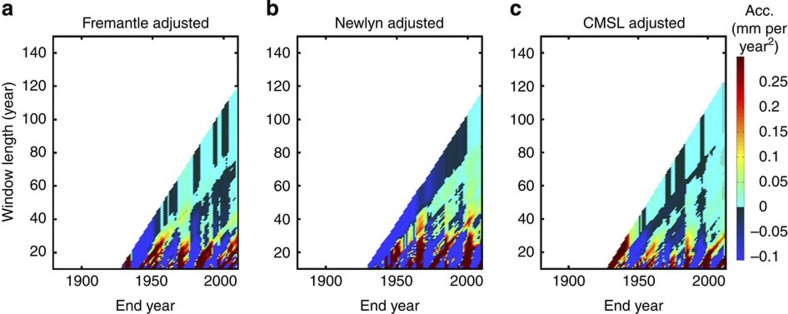
Accelerations for historic sea level records. These records have been adjusted for interannual variability by Calafat and Chambers^27^ (**a**) Fremantle; (**b**) Newlyn; and (**c**) the CMSL, created by averaging their 10 tide gauge records. Hatched area identifies plot regions where accelerations are significantly different from zero (95% confidence interval).

Calafat and Chambers^27^ recently quantified the contribution of natural internal climate variability to sea level changes, using regression models with atmospheric pressure, wind and climate indices as independent variables, with a focus on virtually the same tide gauge sites as we selected. Averaging rate differences from the tide gauge records they selected, they found a statistically significant (90% confidence) acceleration (0.022±0.115 mm per year^2^) between 1952 and 2011, which was unique over the whole period considered in their study (1920–2011). We repeated our analysis using their adjusted tide gauge records. For just their historic records up to 2011, we find (in agreement with them, but using the quadratic model for estimating acceleration rather than rate differences) acceleration statistically different from zero at around 2010 in their equivalent of our CMSL time series (Fig. 7c). We find positive accelerations in individual tide gauge records (Fig. 7a,b), but these are not statistically significant as of 2011 at the 95% confidence level.

**Figure 8:**
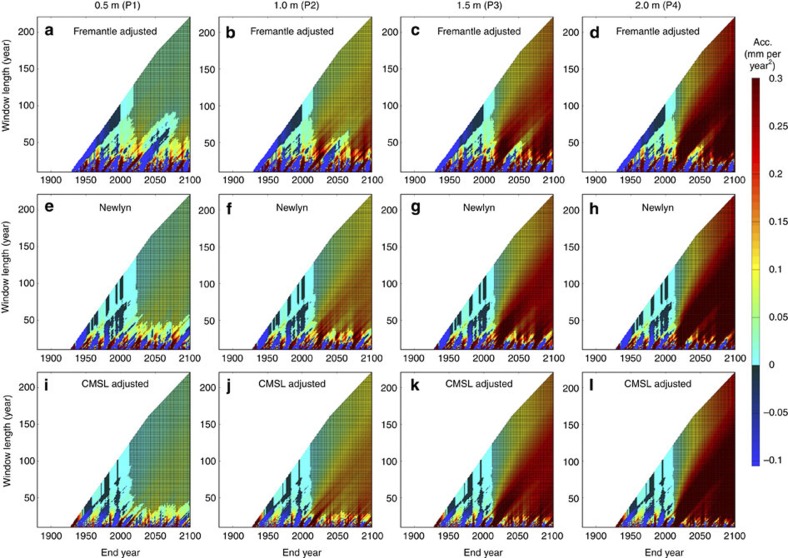
Accelerations for sea level records artificially extended to 2100. These records have been adjusted for interannual variability by Calafat and Chambers^27^ (**a**–**d**) Fremantle to illustrate a site with relatively large interannual variability; (**e**–**h**) Newlyn to illustrate a site with relatively small interannual variability; and (**i**–**l**) the CMSL, created by averaging their 10 tide gauge records. Hatched area highlights plot regions where accelerations are significantly different from zero (95% confidence interval). Note, for each sea level record, the plots show results for just one of the 10,000 randomly generated noise time signals.

Artificially extending the Calafat and Chambers^27^ adjusted tide gauge records to 2100, we detect (Fig. 8) significant accelerations up to two decades earlier than when we used uncorrected tide gauge records. However, even in the largest sea level rise scenario considered here, accelerations significantly different from zero are only identified towards the end of the current decade or early next decade in individual tide gauge records (Fig. 8).

Finally, what would instill confidence in our understanding of whether the rate of sea level rise is increasing is not so much whether an acceleration can be detected, but whether observed changes agree with the expectations from both global climate models and estimated budgets. Church *et al.*^44^ built upon significant recent progress^45^ in understanding the 20th century sea level budget, to explore the relative contributions of different processes to changes in the rate of global sea level rise from 1900–2010. They showed that thermal expansion from the CMIP5 (Coupled Model Intercomparison Project Phase 5) models (used in the latest IPCC projections), combined with estimates of other contributions to sea level rise, are consistent with global sea level observations since around 1950. Furthermore, they found an increase in the rate of global sea level rise from about 1980–2010, which can be explained as the sum of ocean thermal expansion, glacier melt and land-water storage contributions, with the contribution from Antarctic and Greenland ice sheet appearing to be relatively small over this period. Moreover, they concluded that the high rate of rise since 1990 cannot be explained solely as part of internal variability, but instead is a direct response to increased radiative forcing (which was both natural and anthropogenic) and will continue to grow with ongoing emissions.

In summary, we have introduced a novel framework for examining sea level accelerations in a future context, which has allowed us to: evaluate the appropriateness of using certain acceleration detection methods on different sea level data sets; and better assess whether we have the right expectations when it comes to detection of accelerations. In regards to the latter, we have demonstrated the fallacy of arguments that we should by now have observed significantly higher accelerations and linear rates of rise that are significantly larger than past rates. Next, our approach has identified the years (with uncertainties) at which increases in the rate of sea level rise might first be recognized in a statistically significant manner (either with, or without removal of understood components of variability from the records), if not apparent already. It has also allowed us to quantify the magnitudes of accelerations that we would expect to observe at different times throughout the 21st Century, for different sea level projections. In other words, if a particular magnitude of acceleration is detected earlier than what we expect, then this serves as an alert that sea level seems to be following a higher projection. A final major outcome of our analysis is that accelerations might be detected much earlier on local scales if the processes behind internal variability were adequately understood, since this would allow removal of these components from the records.

Considering all this, there is substantial evidence, in both GMSL data sets^2^,^24^ and coastal averaged sea level time series (corrected for internal variability^27^), for the existence and significance of a sustained increase in the rate of sea level rise over the 20th century and early part of the 21st century. In addition, the magnitude of the acceleration currently being observed is consistent with the latest understanding of sea level budgets^45^ and since about 1990 cannot be explained solely as part of internal variability^44^. The public and policy makers might prefer to see evidence of a significant acceleration in their local tide gauge records. However, our results clearly show that it could be several decades before the acceleration detection methods considered here reveal (in a statistically significant sense to 95% confidence) such a discernable acceleration in individual tide gauge records. This is due mainly to the considerable interannual to multidecadal variability evident in sea level at a local scale, and our inability to account fully for all of it at present. Our results imply that if/when the currently understood components of the variability in the records are removed, then accelerations significantly different from zero are likely to become detectable in individual tide gauge records later this decade or early next decade, using the methods considered here.

## Methods

### Data sets

Ten tide gauge records were selected for this analysis (Table 1). Site selection, except for Fremantle, Sydney, Brest, and Den Helder, follows Holgate^33^. Three of his nine sites (Balboa, Cascais and Auckland) have been omitted here because no data were available for recent years. We included Brest because it covers most of the 19th Century, Den Helder to include a long record from the North Sea, and Fremantle and Sydney (Fort Denison) because they are among the longest records in the southern hemisphere. Annual mean sea level values for each site were downloaded from the Permanent Service for Mean Sea Level (PSMSL) ( http://www.psmsl.org). Records were corrected for the effects of glacial isostatic adjustment, using the ICE-5G model results available from the PSMSL. Like Merrifield *et al.*^37^, we emphasize that this does not affect our results, because the vertical land movement rate at each site is assumed constant.

Following previous work^27^,^33^, we created a simple CMSL time series by averaging the 10 tide gauge records (after correcting for glacial isostatic adjustment), using only years for which data were available for at least 4 of the 10 sites. A more densely measured GMSL record was downloaded from http://www.cmar.csiro.au/sealevel/sl_data_cmar.html. The GMSL time series starts in 1880 and contains data up to 2009, so we have restricted our analysis up to that time.

### Creation of sea level records artificially extended to 2100

We then created time series that artificially extend these 12 records to 2100, using four different sea level projections, each superimposed with 10,000 randomly generated noise time series (that is, 480,000 time series in total) to represent future interannual variability (Fig. 2). The first two projections (P1, P2) were based on results from the IPCC’s AR5. The latter two (P3, P4) are based on P2, but were adjusted to reflect projections at the upper end of the scale as suggested by studies using semi-empirical approaches^3^,^4^,^5^,^6^,^7^. We focused on estimates for the low and upper range of the RCP8.5, which correspond approximately to 0.5 and 1 m of sea level rise by 2100, respectively, relative to 1986–2005 (see Table 13.5 of ref. 1). We scaled the upper projection, to create two additional projections, corresponding to 1.5 and 2 m sea level rise over the period. As our historic sea level records have data up to 2009, we only consider the relative projected sea level change for the period from 2010–2100. To do this, we removed the data that pre-date 2010 for the four projection time series and then subtract the level in 2009 from each time series.

We used the Allen and Smith^16^ AR(1) model to generate the future time series of realistic interannual variability. First, Lag-1 autocorrelation and noise variance parameters were individually estimated from each of the 12 de-trended (using a linear rate estimated over the common period 1915–2009) sea level records (Table 1). For each of the 12 records in turn, we then used the AR(1) model, with the autocorrelation and variance parameters estimated from that particular historic record, to randomly generate 10,000 time series, which represent a range of realistic future (2010–2100) interannual variability (Fig. 2).

### Acceleration detection techniques

For each artificially extended record, both quadratic and linear equations were fitted to different periods, and quadratic and linear coefficients and their uncertainty (at 95% confidence interval) were estimated. A least square regression was performed on the annual mean sea level values according to the simple quadratic:


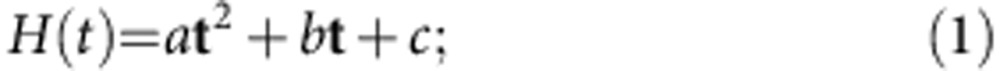


or linear:


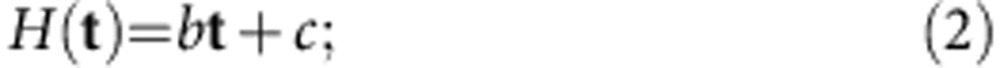


parameterization, where: *a* (mm per year^2^), *b* (mm per year) and *c* (mm) are constants; and **t** is the time in years. In the case of the quadratic function, the acceleration is twice *a* (that is, 2*a*).

We followed the approach of Jevrejeva *et al.*^12^ (and applied recently by Scafetta^26^) and systematically estimated quadratic coefficients for all possible start dates and all possible data lengths (for example, when considering 50-year data lengths on a sea level record starting in 1880, the subsets 1880–1929, 1881–1930 and so on up to 2051–2100 were analysed; when considering 51-year data lengths, the subsets 1880–1930, 1811–1931 and so on up to 2050–2100 were analysed), starting from 10 years (which is the minimum period we use to estimate a quadratic or linear coefficient). Jevrejeva *et al.*^12^,^24^ and Scafetta^26^ plotted results on coloured diagrams, where: the *x* axis was the central year of the period over which the acceleration was calculated; the *y* axis was the data length, in years, of this time period; and the colour represented the estimated acceleration (mm per year^2^). We have adopted this approach. However, when plotting the results, we used the end year of the period on the *x* axis, instead of the central year of the period. We also use a modified colour pallet with a more distinct colour change at zero (than Scafetta^26^), and overlaid the plot with a hatched area to identify plot regions where accelerations are significantly (at 95% confidence interval) different from zero. These changes are to better highlight the year when a significant acceleration is first detected and to identify when accelerations are consistently significant different from zero despite changes in start date.

Following this, we fitted a linear regression model to overlapping periods of different lengths, to each of our artificial time series, and estimated the value and uncertainty (at 95% confidence interval) of the linear coefficient for each period. We assessed lengths of 10-, 20-, 30-, 40- and 50-year periods (for example, for 10-year overlapping periods in the GMSL time series, we analysed the periods 1880–1889, 1881–1890, 1882–1891 and so on up to 2000–2009). We identified, for each of the artificial time series, the end year of the period when the lower 95% confidence limit of the linear rate, for that particular period, was first (and following that, consistently) higher than the upper 95% confidence limits of the linear rates for the historic pre-2010 period; taking into account uncertainty due to future interannual variability as described below.

We took into account two types of uncertainty. First the uncertainty associated with the quadratic and linear coefficient estimates for different periods, which was expressed as a standard error. We recognize that the standard error of a linear rate or acceleration is based on the assumption that each annual mean value is independent and hence underestimates the true error because serial autocorrelation is not taken into account^2^,^46^. Therefore, we reduced the number of degrees of freedom, using the lag-1 autocorrelation of the time series^46^. Confidence intervals (95%) were estimated by multiplying the adjusted standard error by the corresponding *t*-value, considering the reduced number of degrees of freedom.

The second type of uncertainty relates to future interannual variability. We cannot predict the years in the future when the variability is going to be either high or low, and clearly this will influence the end year when an acceleration statistically different from zero is first identified and the end year when a linear rate significantly higher than past rates is identified. Hence, rather than just applying acceleration detection methods to one time series, for each of the four sea level projections artificially extended to 2100; we consider 10,000 time series artificially extended to 2100. Each was generated randomly using the AR(1) model, with autocorrelation and variance parameters, listed in Table 1 and estimated from that particular historic record being used.

We systematically estimated accelerations for all possible start dates and all possible data lengths for each of the 10,000 time series artificially extended to 2100, for each particular sea level record and sea level projection. For each, we then estimated the end year of the period for which the acceleration was estimated when accelerations statistically different from zero consistently become evident in the sea level records (irrespective of start dates). Note that Figs 4, 8 show results for just one of the 10,000 randomly generated noise time signals (for illustration purposes), but the analysis was undertaken on each of the 10,000 time series associated with each of the 12 records and four sea level projections.

For the overlapping linear rate approach, we calculate the end year of the period when the lower 95% confidence limit of the linear rate for that particular period was first higher than the upper 95% confidence limits of the linear rates for the historic pre-2010 period (in all 10,000 time series associated with the particular sea level record and sea level projection considered). Hence, we had 10,000 estimates of the end year when unprecedented rates of rise are first identified, for each record and sea level projection. In Fig. 5, box plots are used to indicate the range of years identified for the 10,000 synthetic time series. On each box, the central mark is the median, the edges of the box are the 25th and 75th percentiles, the whiskers extend to the most extreme data points not considered outliers, and outliers are plotted individually as circles.

### Assumptions

Our analysis included a number of assumptions, which could affect the results presented. We used the sea level projections from IPCC models up to 2100, which slowly accelerate over time (Fig. 2). This obviously is a simplification, which ignores potential abrupt changes that are particularly plausible in the higher 1–2 m projections. Also, in generating the synthetic future part of the time series for the tide gauge locations, we used global average sea level projections, even though the satellite altimetry record reveals that rates of sea level change are highly non-uniform spatially^1^,^2^. Furthermore, we recognize that the model choice for superimposing variability could influence the results. The noise model applied here only accounts for short-term (approximately less than 50 years) variability, while long-term fluctuations present in sea level time series^41^ remain unresolved. However, due to the current lack of knowledge of the latter processes, and for simplicity, we decided to only apply the AR(1) model. Further work could explore more complex models. We also assume that the variability in the future is the same as that observed in the past. Therefore, our main aim cannot be to identify specific years when accelerations are likely to become readily apparent because of these assumptions. Instead, this approach allows us to assess whether we have the right expectations when it comes to the detection of increases in the rate of sea level rise, and whether we are using appropriate detection methods on appropriate data sets. Thus, we aim to bolster the theoretical/statistical underpinning of acceleration detections, which will reduce uncertainties in the debate.

In introducing this novel approach for assessing accelerations, we have just focused on the two main methods that have previously been used to explore increases in the rate of sea level rise. However, the appropriateness of other methods could easily be tested using our methodology. In addition, the assessment could be repeated for different magnitudes of total sea level rise, and different projection profiles.

## Author contributions

I.D.H., T.W., R.M.P., C.B.P. and S.D. conceived the assessment. I.D.H. ran the simulations and produced the figures. I.D.H., T.W. and E.J.R. wrote the manuscript. F.M.C. provided the adjusted sea level time series. All the authors shared ideas, contributed to the interpretation of the results and reviewed the manuscript.

## Additional information

**How to cite this article**: Haigh, I. D. *et al.* Timescales for detecting a significant acceleration in sea level rise. *Nat. Commun.* 5:3635 doi: 10.1038/ncomms4635 (2014).

## Figures and Tables

**Table 1:** Accelerations for the 12 records over their respective record lengths.

**Record**	**Record period**	**Acceleration (mm per year^2^)**	**AR(1) Parameters**	
			**Lag-one autocorrelation**	**Noise variance (mm**^**2**^**)**
Sydney	1915–2009	−0.0048±0.0113	0.35	23.4
Fremantle	1897–2009	−0.0090±0.0137	0.32	45.0
Trieste	1905–2009	−0.0048±0.0095	0.20	30.7
Den Helder	1865–2009	−0.0043±0.0047	0.24	33.6
Newlyn	1915–2009	0.0023±0.0113	0.30	24.9
Brest	1807–2009	0.0096±0.0019	0.25	29.4
New York	1856–2009	0.0077±0.0035	0.26	28.3
Key West	1913–2009	0.0011±0.0105	0.38	23.0
San Diego	1906–2009	−0.0013±0.0095	0.29	28.4
Honolulu	1905–2009	−0.0111±0.0105	0.24	32.2
CMSL	1910–2009	0.0064±0.0056	0.26	11.5
GMSL	1880–2009	0.0099±0.0017	0.60	5.1

Uncertainty reported for the acceleration corresponds to one standard error. Also listed are the two AR(1) parameters estimated for each record, after the record was linearly de-trended over the common period 1915–2009.

**Table 2:** Accelerations for the 12 records for five time periods.

**Record**	**Acceleration (mm per year^2^)**				
	**1880**–**2009**	**1900**–**2009**	**1915**–**2009**	**1930**–**2009**	**1960**–**2009**
Sydney			0.0048±0.0113	−0.0203±0.0164	−0.0151±0.0522
Fremantle			−0.0140±0.0200	0.0067±0.0310	0.1414±0.1034
Trieste			0.0071±0.0131	0.0119±0.0176	0.1334±0.0470
Den Helder	0.0069±0.0059	0.0085±0.0094	0.0175±0.0131	−0.0209±0.0216	0.0377±0.0730
Newlyn			0.0023±0.0113	0.0198±0.0168	0.1253±0.0522
Brest	0.0016±0.0062	−0.0003±0.0096	0.0200±0.0124	0.0340±0.0165	0.1208±0.0520
New York		0.0022±0.0081	−0.0038±0.0115	−0.0093±0.0178	0.0835±0.0631
Key West			−0.0012±0.0111	0.0042±0.0175	−0.0184±0.0541
San Diego			−0.0062±0.0121	−0.0092±0.0192	−0.1036±0.0661
Honolulu			0.0042±0.0130	−0.0017±0.0190	−0.0003±0.0641
CMSL			−0.0017±0.0059	0.0034±0.0089	0.0692±0.0262
GMSL	0.0099±0.0017	0.0118±0.0026	0.0097±0.0037	0.0036±0.0052	0.0286±0.0165

Uncertainty in the acceleration corresponds to one standard error.
